# Correction: theoretical study on the mechanism, chemo- and enantioselectivity of the Ag- *vs.* Rh-catalyzed intramolecular carbene transfer reaction of diazoacetamides

**DOI:** 10.1039/d2ra90074b

**Published:** 2022-07-18

**Authors:** Qingmin Song, Jiayi Wu, Nikolaos V. Tzouras, Yong Wu, Steven P. Nolan

**Affiliations:** Department of Chemistry Northeast Normal University Changchun 130024 China wuy651@nenu.edu.cn; Department of Chemistry and Center for Sustainable Chemistry, Ghent University Krijgslaan 281, S3 Ghent 9000 Belgium Steven.Nolan@UGent.be

## Abstract

Correction for ‘Theoretical study on the mechanism, chemo- and enantioselectivity of the Ag- *vs.* Rh-catalyzed intramolecular carbene transfer reaction of diazoacetamides' by Qingmin Song *et al.*, *RSC Adv.*, 2022, **12**, 18197–18208, https://doi.org/10.1039/D2RA01298G.

The authors regret that the name of one of the authors (Nikolaos V. Tzouras) was shown incorrectly in the original article. The corrected author list is as shown above.

The Royal Society of Chemistry apologises for these errors and any consequent inconvenience to authors and readers.

## Supplementary Material

